# Evaluation of the causal effects of immune cells on ischemic stroke: a Mendelian randomization study

**DOI:** 10.3389/fimmu.2024.1374350

**Published:** 2024-05-24

**Authors:** Kunyu Wang, Beilin Zhang, Min Li, Hanying Duan, Zhuoya Jiang, Su Gao, Jing Chen, Shaokuan Fang

**Affiliations:** Department of Neurology, Neuroscience Research Center, The First Hospital of Jilin University, Changchun, China

**Keywords:** immune cells, ischemic stroke, Mendelian randomization, causal effect, single nucleotide polymorphism

## Abstract

**Background:**

Ischemic stroke (IS) is a cerebrovascular disease caused by various factors, and its etiology remains inadequately understood. The role of immune system dysfunction in IS has been increasingly recognized. Our objective was to evaluate whether circulating immune cells causally impact IS risk.

**Methods:**

We conducted two-sample Mendelian randomization analyses to evaluate the causal effects of 731 immune cell traits on IS, utilizing publicly available genome-wide association studies (GWAS) summary statistics for 731 immune cell traits as exposure data, and two GWAS statistics for IS as outcome data. A set of sensitivity analyses, including Cochran’s Q test, *I* ^2^ statistics, MR-Egger intercept test, MR-PRESSO global test, and leave-one-out sensitivity analyses, were performed to assess the robustness of the results. Additionally, meta-analyses were conducted to combine the results from the two different IS datasets. Finally, we extracted instrumental variables of immune cell traits with causal effects on IS in both IS datasets for SNP annotation.

**Results:**

A total of 41 and 35 immune cell traits were identified to have significant causal effects on IS based on two different IS datasets, respectively. Among them, the immune cell trait *CD62L^-^ plasmacytoid Dendritic Cell AC* and *CD4^+^ CD8^dim^ T cell%leukocyte* respectively served as risk factor and protective element in both IS datasets. The robustness of the causal effects was confirmed through the sensitivity analyses. The results of the meta-analyses further support the causal effects of *CD62L^-^ plasmacytoid Dendritic Cell AC* (pooled OR=1.030, 95%CI: 1.011–1.049, *P*=0.002) and *CD4^+^ CD8^dim^ T cell%leukocyte* (pooled OR=0.959, 95%CI: 0.935–0.984, *P*=0.001). Based on these two immune cell traits, 33 genes that may be related to the causal effects were mapped.

**Conclusions:**

Our study demonstrated the potential causal effects of circulating immune cells on IS, providing valuable insights for future studies aimed at preventing IS.

## 1 Introduction

Stroke ranks as the second most common cause of death and the third leading cause of death and disability combined worldwide, causing a huge burden to the economy and society (1). Ischemic stroke (IS) is the predominant type of stroke, resulting from a blockage in the blood supply to the brain and clinically manifested by transient or permanent brain dysfunction. In 2020, the global incidence of stroke was 11.71 million people, with IS accounting for approximately 65% of all cases (2). Current treatments for IS rely on rapidly clearing the blockage through thrombolysis or mechanical approaches, and the treatment effectiveness closely tied to the intervention time window (3). The pathogenesis of IS is complex, often resulting from the combined effect of multiple factors. Hypertension, hyperlipidemia, atrial fibrillation, cigarette smoking, excessive alcohol consumption, and diabetes mellitus are well-established risk factors for IS (4–6). However, these traditional risk factors can only partially explain the risk of IS. Therefore, accurately identifying novel IS-related risk factors has become crucial for its prevention and treatment.

The role of inflammation and immune system dysfunction in IS has been increasingly recognized (7). Immune cells, crucial components of the immune system, circulate in the bloodstream or reside within tissues. A transcriptomic study revealed the involvement of immune cells in IS, demonstrating significant differences in peripheral blood immune cells of IS patients compared to the normal control group (8). Studies have indicated that both acute and chronic inflammation in IS is primarily linked to immune cells such as B cells, T cells, monocytes, neutrophils (7, 9). A complex interdependent relationship exists among different types of immune cells in IS. They not only collaborate to clear necrotic brain tissue but may also trigger an inflammatory response, leading to damage of healthy neurons (7, 9). However, a significant portion of the existing evidence is derived from observational studies, which might be constrained by confounding factors and reverse causality. This means that while specific changes in immune cells may be observed in associated with IS, it cannot be determined whether the changes in immune cells are a direct cause of IS or a result of it.

Mendelian randomization (MR) is a widely used analytical method aimed at investigating potential causal impacts of exposures on outcomes using data obtained from genome-wide association studies (GWAS) (10). MR effectively reduces the influence of confounding factors and avoids the issue of reverse causation commonly encountered in observational studies, as allelic variants are randomly allocated and fixed at conception (11).

To the best of our knowledge, the causal association between a broad range of immune cell traits and IS has not been established using MR. To address this gap, based on the available GWAS data on peripheral blood immune cells and IS, two-sample MR analyses were performed to explore the causal links of 731 types of immune cell trait on IS risk.

## 2 Materials and methods

### 2.1 Study design

**Figure 1:**
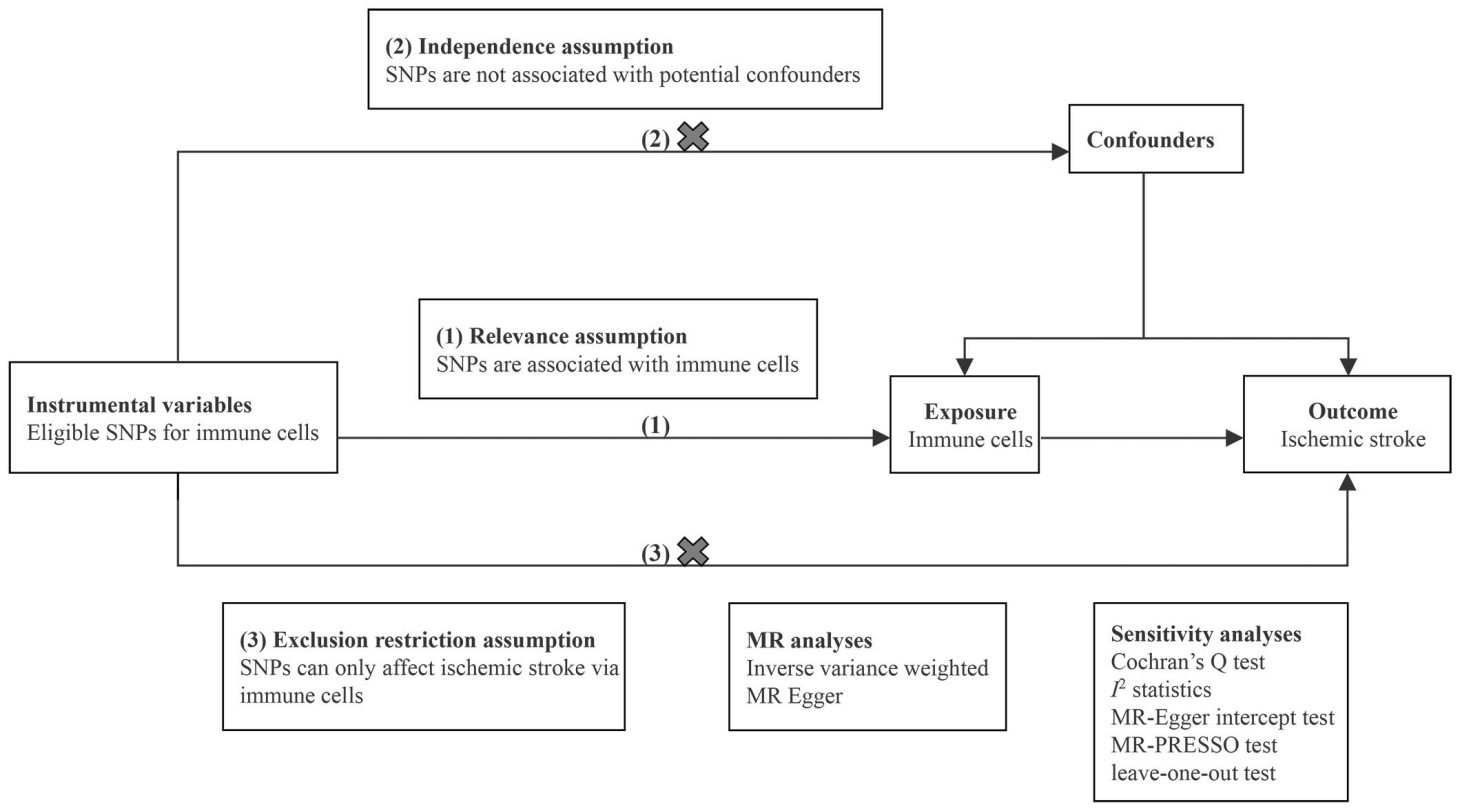
Schematic diagram of the MR study.

We conducted two-sample MR analyses to evaluate the causal effects of 731 immune cell traits on the risk of IS. Each immune cell trait served as an exposure variable, and IS served as the outcome variable. Eligible single nucleotide polymorphisms (SNPs) that represented immune cell traits were employed as instrumental variables (IVs). In the MR analysis, adherence to three fundamental assumptions is essential: (1) relevance assumption: IVs are strongly associated with immune cells; (2) independence assumption: IVs are independent of potential confounders; and (3) exclusion restriction assumption: IVs affect IS only via immune cells (12). Sensitivity analyses were performed to ensure the robustness of the results. The design of MR analysis is illustrated in **Figure 1**. All studies included in our analysis received approval from the relevant institutional review boards.

### 2.2 Data sources

We obtained the GWAS data for immune cell traits and IS from the IEU Open GWAS project website (https://gwas.mrcieu.ac.uk/datasets). For the exposure data, we used the GWAS summary statistics for a total of 731 immune cell traits derived from a cohort of 3,757 normal Europeans (13). These immune cell traits were classified into four groups: absolute cell counts (AC) (n=118), relative cell counts (RC) (n=192), median fluorescence intensities (MFI) (n=389), and morphological parameters (MP) (n=32). Specifically, AC, RC, and MFI contain TBNK (T cell, B cell, natural killer cell), Treg, maturation stages of T cell, dendritic cell (DC), B cell, monocyte, and myeloid cell panels, while MP contains DC and TBNK panels. To ensure comparability in ancestry, outcome data for IS were extracted from two GWAS statistics of Europeans with the largest sample sizes, consisting of 11,929 cases/472,192 controls (GWAS ID: ebi-a-GCST90018864; designated as the discovery dataset) (14) and 34,217 cases/406,111 controls (GWAS ID: ebi-a-GCST005843; designated as the validation dataset) (15), respectively. Detailed information of datasets is presented in **Table 1**.

**Table 1:** Detailed information of datasets.

GWAS ID	Phenotype	Sample size	Case	Control	Ancestry
ebi-a-GCST90001391– ebi-a-GCST90002121	Immune cell traits	–	–	–	European
ebi-a-GCST90018864(discovery dataset)	Ischemic stroke	484,121	11,929	472,192	European
ebi-a-GCST005843(validation dataset)	Ischemic stroke	440,328	34,217	406,111	European

### 2.3 Selection of IVs

To ensure the authenticity and reliability of IVs, a set of quality control measures was implemented. Firstly, in accordance with the recent studies (16, 17), the significance level of IVs for each immune cell trait was set at 1×10^−5^. Secondly, to obtain independent IVs, the linkage disequilibrium r^2^ threshold was set to 0.001 within a 10,000kb distance based on the reference panel of 1000 Genomes Project (18). Thirdly, SNPs significantly correlated with confounders such as arterial hypertension and diabetes mellitus as previously reported (19) were excluded using PhenoScanner (http://www.phenoscanner.medschl.cam.ac.uk/) to preliminarily mitigate the effect of horizontal pleiotropy (**Supplementary Table 1**). Fourthly, to avoid bias from weak instruments, only IVs with *F*-statistics greater than 10 were considered as strong instruments. Finally, the GWAS data for each immune cell trait dataset and each IS dataset were harmonized with the selected IVs.

### 2.4 Statistical analyses

To evaluate the potential causal effects of 731 immune cell traits on IS, inverse variance weighting (IVW) as the primary method and MR-Egger as the supplementary method were conducted. The obtained results were visualized using scatter plots. Subsequently, a range of sensitivity analyses were performed to assess the robustness of the results. Cochran’s Q test and *I* ^2^ statistics were used to detect the heterogeneity among IVs. MR-Egger intercept test was utilized to evaluate the presence of horizontal pleiotropy (20). The MR-PRESSO global test, known for higher statistical power, was also employed to further examine possible horizontal pleiotropy (21). Additionally, leave-one-out sensitivity analyses were performed to determine whether an individual SNP could influence the bias of causal estimate. Finally, to facilitate the integration of results from the two different IS datasets, meta-analyses were conducted to consolidate the findings. The analyses were carried out using the packages TwoSampleMR (version 0.5.6), MR-PRESSO (version 1.0), and meta (version 6.5) in R (version 4.1.0). Detailed procedure code is provided in **Supplementary Materials**.

### 2.5 SNP annotation

An rs-codes of SNP converter g:SNPense was utilized for SNP annotation (22). g:SNPense is an online tool for mapping human SNP identifiers to their corresponding genes and providing their predicted variant effects, with the Ensembl Variation data. Mapping is only available for SNPs which overlap with at least one Ensembl gene.

## 3 Results

### 3.1 Selection of IVs

Following stringent quality control measures, we identified 2 to 729 independent IVs for different immune cell traits. The *F*-statistics for these IVs ranged from 19.548 to 2435.818, indicating a lack of potential bias from weak instruments. Comprehensive details about the IVs, including rs-codes, effect allele, other allele, beta value, standard error, *P*-value, and other information, are systematically summarized in **Supplementary Table 2**.

### 3.2 MR analyses

**Figure 2:**
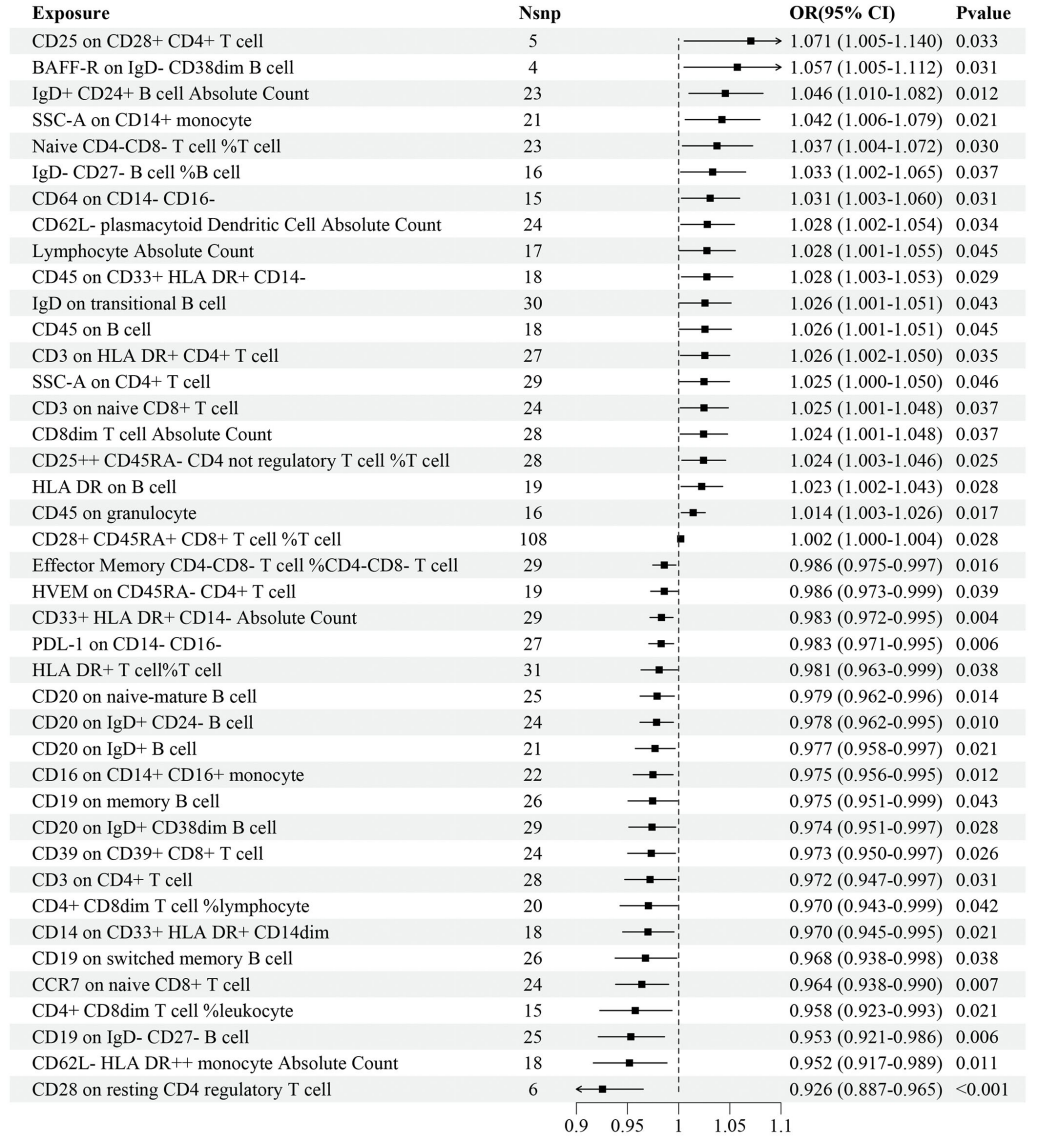
Forest plot for the causal effects of immune cell traits on IS risk derived from inverse variance weighted based on discovery dataset. OR, odds ratio; CI, confidence interval.

Regarding the discovery dataset, the results of the IVW analyses revealed 41 immune cell traits exhibiting significant causal associations with IS risk, including 11 in the B cell panel, 10 in the TBNK panel, 7 in the Treg panel, 5 in the maturation stages of T cell panel, 3 in the monocyte panel, 3 in the myeloid cell panel, and 2 in the DC panel (**Figure 2**). A total of 20 immune cell traits, such as *CD25 on CD28^+^ CD4^+^ T cell* (OR=1.071, 95%CI: 1.005–1.140, *P*=0.033), *BAFF-R on IgD^-^ CD38^dim^ B cell* (OR=1.057, 95%CI: 1.005–1.112, *P*=0.031), and *IgD^+^ CD24^+^ B cell AC* (OR=1.046, 95%CI: 1.010–1.082, *P*=0.012), were found to significantly increase the risk of IS. Conversely, 21 immune cell traits, such as *CD28 on resting CD4 regulatory T cell* (OR=0.926, 95%CI: 0.887–0.965, *P*<0.001), *CD62L^-^ HLA DR^++^ monocyte AC* (OR=0.952, 95%CI: 0.917–0.989, *P*=0.011), and *CD19 on IgD^-^ CD27^-^ B cell* (OR=0.953, 95%CI: 0.921–0.986, *P*=0.006), significantly decreased the risk of IS (**Figure 2**).

**Figure 3:**
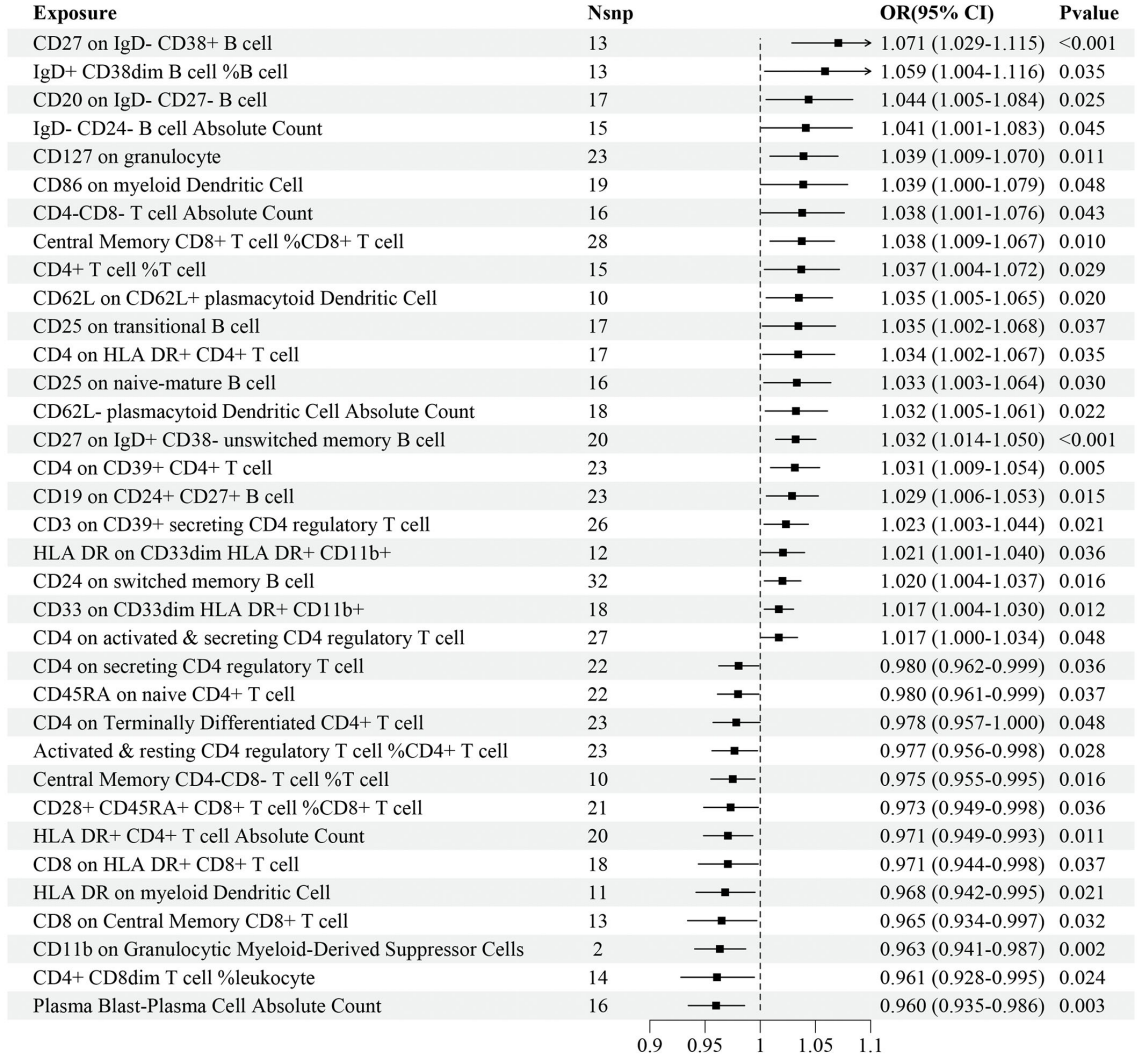
Forest plot for the causal effects of immune cell traits on IS risk derived from inverse variance weighted based on validation dataset. OR, odds ratio; CI, confidence interval.

Regarding the validation dataset, the results of the IVW analyses demonstrated 35 immune cell traits exhibiting significant causal associations with IS risk, of which 10 were in the B cell panel, 5 in the TBNK panel, 7 in the Treg panel, 6 in the maturation stages of T cell panel, 3 in the myeloid cell panel, and 4 in the DC panel (**Figure 3**). Among these immune cell traits, 22 of them, such as *CD27 on IgD^-^ CD38^+^ B cell* (OR=1.071, 95%CI: 1.029–1.115, *P*<0.001), *IgD^+^ CD38^dim^ B cell%B cell* (OR=1.059, 95%CI: 1.004–1.116, *P*=0.035), and *CD20 on IgD^-^ CD27^-^ B cell* (OR=1.044, 95%CI: 1.005–1.084, *P*=0.025), were positively associated with the risk of IS. On the contrary, 13 immune cell traits, such as *Plasma Blast-Plasma Cell AC* (OR=0.960, 95%CI: 0.935–0.986, *P*=0.003), *CD4^+^ CD8^dim^ T cell%leukocyte* (OR=0.961, 95%CI: 0.928–0.995, *P*=0.024), and *CD11b on Granulocytic Myeloid-Derived Suppressor Cells* (OR=0.963, 95%CI: 0.941–0.987, *P*=0.002), were negatively associated with the risk of IS (**Figure 3**). Details of MR analyses, including the results estimated by MR-Egger, are summarized in **Supplementary Table 3**.

Based on the results of MR analyses of the discovery and validation datasets, we found that *CD62L^-^ plasmacytoid Dendritic Cell AC* and *CD4^+^ CD8^dim^ T cell%leukocyte* were linked with the susceptibility to IS in both datasets, serving as a risk factor and a protective element, respectively (**Table 2**). Scatter plots illustrating the causal effects of *CD62L^-^ plasmacytoid Dendritic Cell AC* and *CD4^+^ CD8^dim^ T cell%leukocyte* in different IS datasets are presented in **Supplementary Figure 1**.

**Table 2:** Common causal immune cell traits based on two ischemic stroke datasets.

GWAS ID	Immune cell trait	OR (95%CI)	*P*-value
ebi-a-GCST90018864 (discovery dataset)	CD62L^-^ plasmacytoid Dendritic Cell Absolute Count	1.028(1.002-1.054)	0.0337
	CD4^+^ CD8^dim^ T cell%leukocyte	0.958(0.923-0.993)	0.0210
ebi-a-GCST005843 (validation dataset)	CD62L^-^ plasmacytoid Dendritic Cell Absolute Count	1.032(1.005-1.061)	0.0222
	CD4^+^ CD8^dim^ T cell%leukocyte	0.961(0.928-0.995)	0.0236

### 3.3 Sensitivity analyses

The results of sensitivity analyses confirmed the robustness of the causal associations (**Table 3**). When *I*² statistics>50% or the *P*-value for Cochran’s Q test<0.05, heterogeneity among IVs needs to be considered. No evidence of heterogeneity was detected in our results. Visualized funnel plots are presented in **Supplementary Figure 2**. Neither the Egger intercept test nor the MR-PRESSO global test identified significant horizontal pleiotropy, except for the Egger intercept test for *CD62L^-^ plasmacytoid Dendritic Cell AC* in the discovery dataset (*P*=0.0022). It is worth noting that, compared to the Egger intercept test, the MR-PRESSO global test demonstrates higher statistical power (21). Therefore, it is justifiable to prioritize the results of the MR-PRESSO global test. However, considering the potential presence of horizontal pleiotropy, we conducted MR-Egger causal estimation to complement the MR analysis, which can identify and adjust for horizontal pleiotropy (20). The MR-Egger estimation also revealed a consistent causal effect for *CD62L^-^ plasmacytoid Dendritic Cell AC* on IS (OR=1.085, 95%CI: 1.045–1.126, *P*<0.001) (**Supplementary Table 3**), aligning with the IVW result in the discovery dataset, demonstrating the reliability of the finding. The results of the leave-one-out sensitivity analyses demonstrated that no single SNP could significantly influence the causal estimates (**Supplementary Figure 3**).

**Table 3:** Evaluation of heterogeneity and horizontal pleiotropy using different methods.

GWAS ID	Immune cell trait	Heterogeneity			Horizontal pleiotropy			
		*I* ^2^(%)	Cochran’s Q	*P*-value	Egger intercept	SE	*P*-value	MR-PRESSO *P*-value
ebi-a-GCST90018864 (discovery dataset)	CD62L^-^ plasmacytoid Dendritic Cell Absolute Count	27	31.5587	0.1097	-0.0169	0.0049	0.0022	0.0680
	CD4^+^ CD8^dim^ T cell%leukocyte	0	10.9666	0.6887	-0.0031	0.0087	0.7240	0.6740
ebi-a-GCST005843 (validation dataset)	CD62L^-^ plasmacytoid Dendritic Cell Absolute Count	15	20.0553	0.2714	-0.0084	0.0056	0.1486	0.2550
	CD4^+^ CD8^dim^ T cell%leukocyte	4	13.4926	0.4105	-0.0046	0.0077	0.5659	0.4770

### 3.4 Meta-analyses

**Figure 4:**
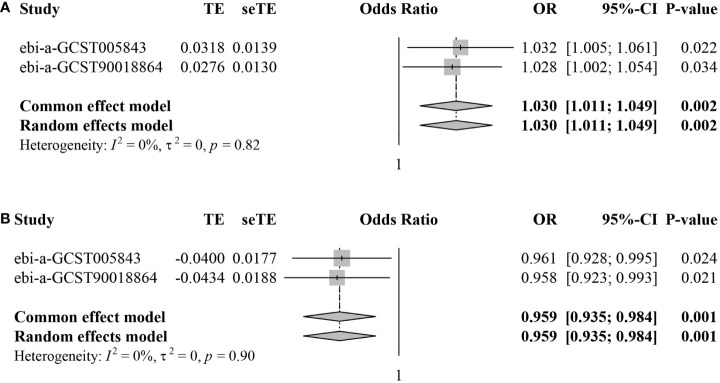
Forest plots for meta-analyses of MR estimates in two IS datasets. **(A)** *CD62L^-^ plasmacytoid Dendritic Cell AC*. **(B)** *CD4^+^ CD8^dim^ T cell%leukocyte*.

Subsequently, we conducted meta-analyses to combine the MR estimates from the two different datasets. For *CD62L^-^ plasmacytoid Dendritic Cell AC*, the meta-analysis results indicated that an increase in *CD62L^-^ plasmacytoid Dendritic Cell AC* led to a higher risk of IS (pooled OR=1.030, 95%CI: 1.011–1.049, *P*=0.002) without any heterogeneity observed (*I* ^2^ = 0.0%, τ^2^ = 0.0%, *P*=0.82) (**Figure 4A**). Regarding *CD4^+^ CD8^dim^ T cell%leukocyte*, the meta-analysis results showed that an increase in this trait decreased the risk of IS (pooled OR=0.959, 95%CI: 0.935–0.984, *P*=0.001) without any heterogeneity observed (*I* ^2^ = 0.0%, τ^2^ = 0.0%, *P*=0.90) (**Figure 4B**).

### 3.5 SNP annotation

We annotated the SNPs as IVs of the immune cell traits of *CD62L^-^ plasmacytoid Dendritic Cell AC* and *CD4^+^ CD8^dim^ T cell%leukocyte*. A total of 33 Ensembl genes were mapped using g:SNPense (**Table 4**). These identified genes may be relevant to the causal effect of *CD62L^-^ plasmacytoid Dendritic Cell AC* and *CD4^+^ CD8^dim^ T cell%leukocyte* on IS risk.

**Table 4:** SNP annotation of immune cell trait instrumental variables.

Immune cell trait	SNP	Chr	Start	End	Strand	Gene id	Gene name
CD62L^-^ plasmacytoid Dendritic Cell Absolute Count	rs116054627	2	50941360	50941360	+	ENSG00000179915	NRXN1
	rs11659751	18	58069491	58069491	+	ENSG00000049759	NEDD4L
	rs116894787	10	12198107	12198107	+	ENSG00000151465	CDC123
	rs118054784	8	129646438	129646438	+	ENSG00000229140	CCDC26
	rs12061996	1	167845147	167845147	+	ENSG00000143199	ADCY10
	rs13201703	6	22921242	22921242	+	ENSG00000233358	ENSG00000233358
	rs1391986	5	52775477	52775477	+	ENSG00000248898	PELO-AS1
	rs1472757	5	6455759	6455759	+	ENSG00000215218	UBE2QL1
	rs150918748	22	43050384	43050384	+	ENSG00000100271, ENSG00000230319	TTLL1, TTLL1-AS1
	rs16957038	13	99889998	99889998	+	ENSG00000125246, ENSG00000286757	CLYBL, ENSG00000286757
	rs170697		-1	-1			
	rs17224524	2	181481419	181481419	+	ENSG00000115232	ITGA4
	rs191779135	4	81581203	81581203	+	ENSG00000138670	RASGEF1B
	rs4462104		-1	-1			
	rs4928176		-1	-1			
	rs56374915		-1	-1			
	rs61936377		-1	-1			
	rs6535445		-1	-1			
	rs7114664	11	22771455	22771455	+	ENSG00000148935	GAS2
	rs72716416	8	130980984	130980984	+	ENSG00000155897	ADCY8
	rs75472065	3	77027522	77027522	+	ENSG00000185008	ROBO2
	rs9403901		-1	-1			
	rs9502274	6	566060	566060	+	ENSG00000112685	EXOC2
	rs9900969	17	78198408	78198408	+	ENSG00000183077	AFMID
CD4^+^ CD8^dim^ T cell%leukocyte	rs10858526		-1	-1			
	rs10880825	12	45598113	45598113	+	ENSG00000257657	ENSG00000257657
	rs114200183	2	96684492	96684492	+	ENSG00000249715	FER1L5
	rs150052968		-1	-1			
	rs150339178	2	85420783	85420783	+	ENSG00000152292, ENSG00000286011	SH2D6, ENSG00000286011
	rs17530643	1	83003581	83003581	+	ENSG00000230817	LINC01362
	rs2801996	1	89907158	89907158	+	ENSG00000171492, ENSG00000271949	LRRC8D, ENSG00000271949
	rs28521508	15	83463237	83463237	+	ENSG00000140600	SH3GL3
	rs35290870	2	86795980	86795980	+	ENSG00000153563	CD8A
	rs4959028		-1	-1			
	rs511713	11	128229204	128229204	+	ENSG00000272575	LINC02098
	rs66907047	10	26597320	26597320	+	ENSG00000236894	LINC03028
	rs78510378	7	155207215	155207215	+	ENSG00000287865	ENSG00000287865
	rs79131379	6	33165263	33165263	+	ENSG00000204248	COL11A2
	rs903881	16	16112185	16112185	+	ENSG00000103222	ABCC1

## 4 Discussion

Understanding the impact of immune cells on IS will provide valuable insights into the role of inflammation and immune system dysfunction in the onset and progression of IS. Recent research has brought attention to the noteworthy influence of immunity on IS risk, as evidenced by elevated levels of inflammatory markers in the bloodstream, such as interleukin-6 (23), monocyte chemotactic protein-1 (24), and C-reactive protein (25), as well as a rise in total white blood cell count (26–28) and neutrophil count (27, 28). However, due to the inherent limitations of observational studies (29), these investigations can only establish the involvement of inflammation and immune cells in the development of IS but cannot offer reliable proof of causality. Given the methodological advantages of MR analysis in causal inference (11), this study’s evaluation of the causal effects of immune cells on the risk of IS may be more dependable than previous observational studies. In our study, utilizing large-scale publicly available genetic data, we conducted two-sample MR analyses to explore genetic evidence supporting causal associations between immune cell traits and IS. The results of this study demonstrated that 41 and 35 immune cell traits had significant causal effects on IS based on the discovery and validation dataset, respectively. Furthermore, the immune cell traits *CD62L^-^ plasmacytoid Dendritic Cell AC* and *CD4^+^ CD8^dim^ T cell%leukocyte* were significant in both the discovery and validation datasets. In addition, the meta-analyses, combining the MR estimates, further confirm that *CD62L^-^ plasmacytoid Dendritic Cell AC* and *CD4^+^ CD8^dim^ T cell%leukocyte* have causal effects on IS.

DCs play a crucial role in initiating and coordinating the immune response as professional antigen-presenting cells, which can be classified into inflammatory DCs, Langerhans cells, conventional DCs, and plasmacytoid DCs (30). The role of DCs has not been sufficiently investigated in the context of IS. Regarding clinical study, compared with healthy individuals, the number of circulating plasmacytoid DC precursors was transiently reduced in IS patients, while a dense infiltration of plasmacytoid DCs was observed in the infarcted brain, indicating the potential recruitment of plasmacytoid DC precursors from blood into the infarcted brain (31). Using a murine model of experimental IS, Barbara et al. observed an induction of DC migration and maturation under ischemic conditions, and inhibiting DC function can reduce the infarct area and improve neurological function scores (32). The adhesion molecule CD62L^-^ DCs are considered immature with relatively low migratory capability (13). According to our research, elevated levels of *CD62L^-^ plasmacytoid Dendritic Cell AC* can increase the risk of IS. Self-DNA released from dying cells can activate neutrophils to release the DNA-binding antimicrobial peptide LL37 (known as Cramp in mice), which in can subsequently convert self-DNA into a trigger for plasmacytoid DC activation through Toll-like receptor 9, leading to the production of large amounts of interferon-α (33, 34). The mechanism of plasmacytoid DC activation by self-DNA is closely related to the occurrence and progression of atherosclerosis and diabetes (35, 36). Both atherosclerosis and diabetes are intricately connected to the onset and development of IS (4, 5), which may partly explain why *CD62L^-^ plasmacytoid Dendritic Cell AC* could serve as a risk factor for IS.

In the realm of immune components, T cells are particularly significant due to their potency in both innate and adaptive immune responses. They are crucially involved in post-stroke inflammation, primarily through the release of inflammatory cytokines and their intricate interplay with other cells, thereby amplifying the cascade of inflammation (37, 38). It has been reported that IS induced a dramatic and immediate loss of circulating T cells within 12 hours after onset (39). However, in the infarcted brain samples of IS patients, T cell numbers have been shown to increase for at least 3 months (40). A recent study demonstrated that the decrease in the percentage of circulating CD4^+^ naïve T cells is a risk factor for IS in patients on hemodialysis (41). In our study, *CD4^+^ CD8^dim^ T cell%leukocyte* in TBNK panel was shown to be significantly associated with decreased risk of IS. Interleukin-10 plays a significant role in regulating pro-inflammatory cytokines and exerting immunomodulatory and neuroprotective effects in the context of IS (42). In a murine model of experimental IS, Dan et al. demonstrated that the adoptive transfer of interleukin-10-producing CD4^+^ T cells resulted in a reduction in ischemic infarct size (43). Further exploration is needed to elucidate the mechanisms underlying the involvement of *CD4^+^ CD8^dim^ T cell%leukocyte* in the occurrence and progression of IS.

We identified 33 genes that may be associated with the causal effect of *CD62L^-^ plasmacytoid Dendritic Cell AC* and *CD4^+^ CD8^dim^ T cell%leukocyte* on IS risk by SNP annotation. Among these genes, NEDD4L (44) and ABCC1 (45) have been reported to be involved in IS through non-immune mechanisms. NEDD4L deletion can exacerbate ischemic brain damage by diminishing α-Synuclein polyubiquitination (44). ABCC1 is downregulated in response to IS, which could be reversed by the deletion of apolipoprotein E (45).

There are several limitations of the present MR study. First, due to the lack of detailed individual information, we could not delve deeper into the causal effects of immune cell traits on subgroups of the population. Second, since the dataset solely represents a European population, caution must be exercised when extrapolating the findings to other ethnic groups, necessitating additional scrutiny. Third, all causal effects uncovered through our MR study were derived from IVs at a relatively loose threshold, which may potentially affect the precision of the results. However, considering all *F*-statistics were greater than 10, it appears unlikely that weak IVs could have influenced our findings.

In summary, our results could offer novel perspectives on the causal connections between immune cell traits and IS, and highlight the intricate interactions between the immune system and IS. The findings indicate that *CD62L^-^ plasmacytoid Dendritic Cell AC* and *CD4^+^ CD8^dim^ T cell%leukocyte* hold potential as biomarkers for IS risk, which could facilitate earlier diagnosis and more effective treatment options. Furthermore, we call for experimental research to explore the underlying mechanisms linking identified immune cell traits to the risk of IS.

## Data availability statement

The original contributions presented in the study are included in the article/**Supplementary Material**. Further inquiries can be directed to the corresponding author.

## Author contributions

KW: Formal analysis, Visualization, Writing – original draft. BZ: Conceptualization, Supervision, Writing – review & editing. ML: Formal analysis, Visualization, Writing – review & editing. HD: Formal analysis, Visualization, Writing – review & editing. ZJ: Formal analysis, Visualization, Writing – review & editing. SG: Formal analysis, Visualization, Writing – review & editing. JC: Formal analysis, Visualization, Writing – review & editing. SF: Conceptualization, Funding acquisition, Supervision, Writing – review & editing.
