# Supplementary material for: Evaluation of the causal effects of immune cells on ischemic stroke: a Mendelian randomization study
Source: Front Immunol. 2024 May 24;15:1374350. doi: 10.3389/fimmu.2024.1374350 (PMC11157000; doi:10.3389/fimmu.2024.1374350)
Supplement: Supplementary file 1 [file DataSheet_1.pdf]

Supplementary Material

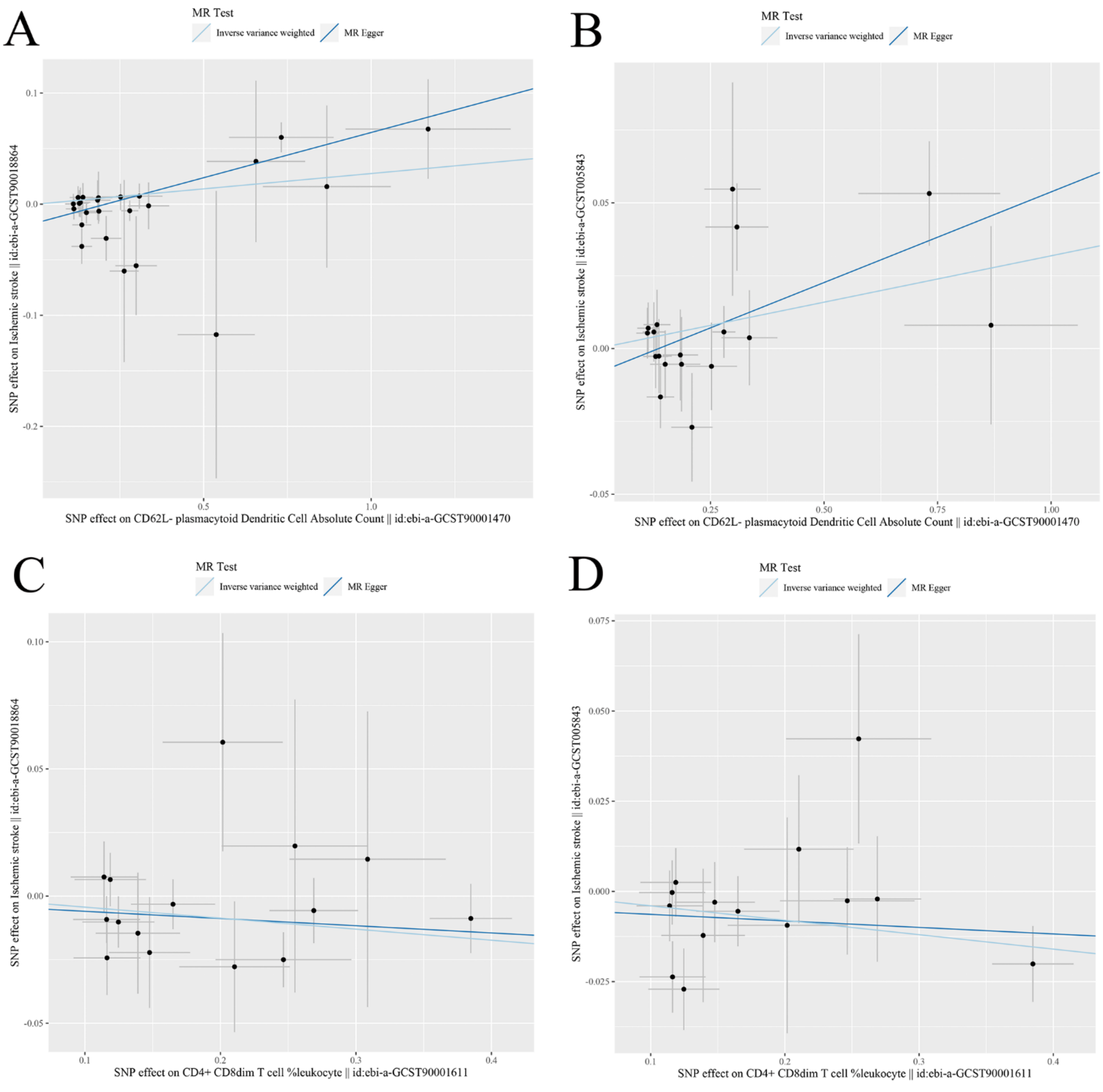

Supplementary Figure 1. Scatter plots for the causal effects of immune cell traits on IS risk. **(A)** *CD62L- plasmacytoid Dendritic Cell Absolute Count* on IS risk based on discovery dataset. **(B)** *CD62L- plasmacytoid Dendritic Cell Absolute Count* on IS risk based on validation dataset. **(C)**

CD4+ CD8dim T cell %leukocyte on IS risk based on discovery dataset. **(D)** *CD4+ CD8dim T cell %leukocyte* on IS risk based on validation dataset.

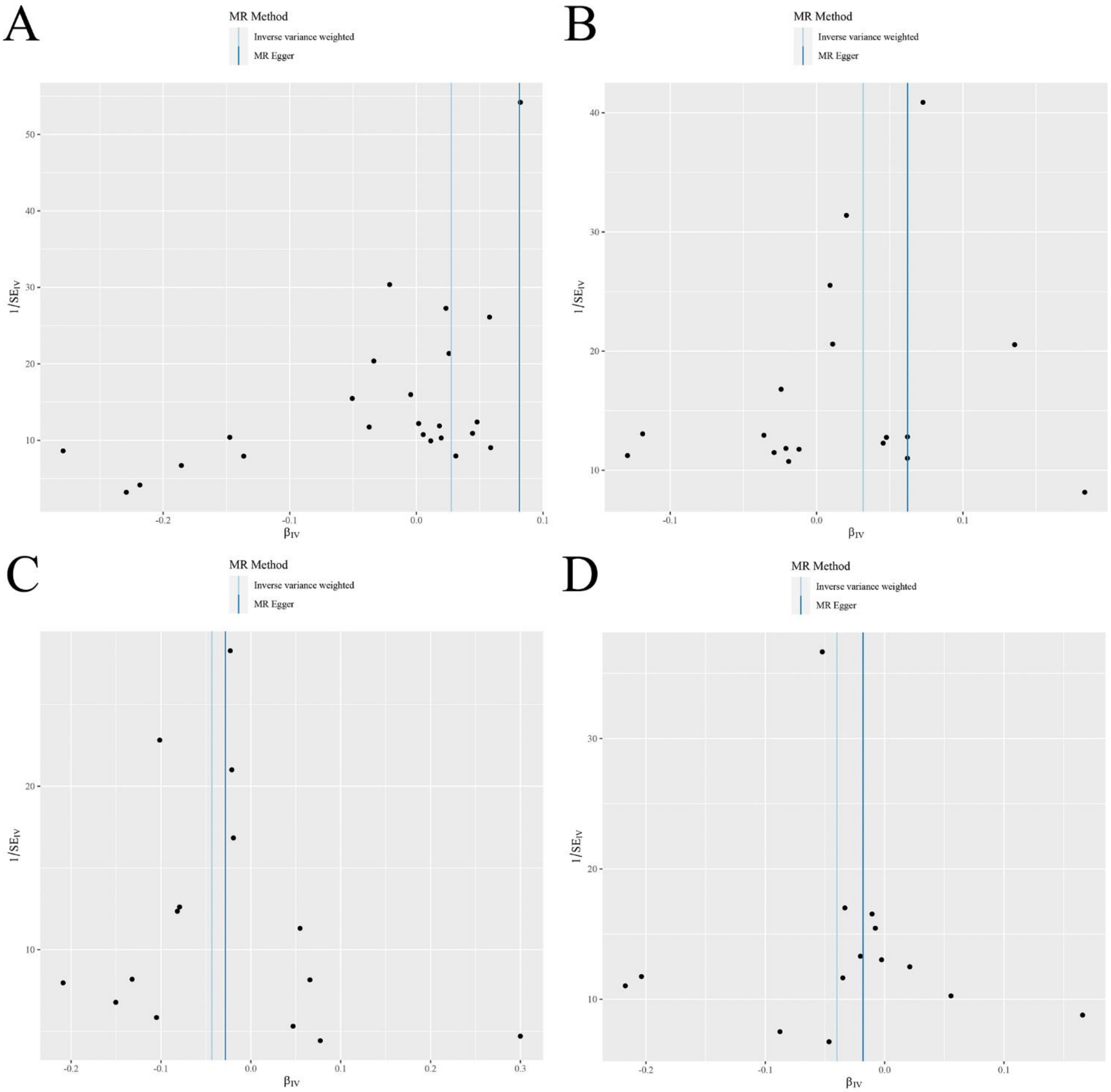

Supplementary Figure 2. Funnel plots for the causal effects of immune cell traits on IS risk. **(A)** *CD62L- plasmacytoid Dendritic Cell Absolute Count* on IS risk based on discovery dataset. **(B)** *CD62L- plasmacytoid Dendritic Cell Absolute Count* on IS risk based on validation dataset. **(C)** *CD4+ CD8dim T cell %leukocyte* on IS risk based on discovery dataset. **(D)** *CD4+ CD8dim T cell %leukocyte* on IS risk based on validation dataset.

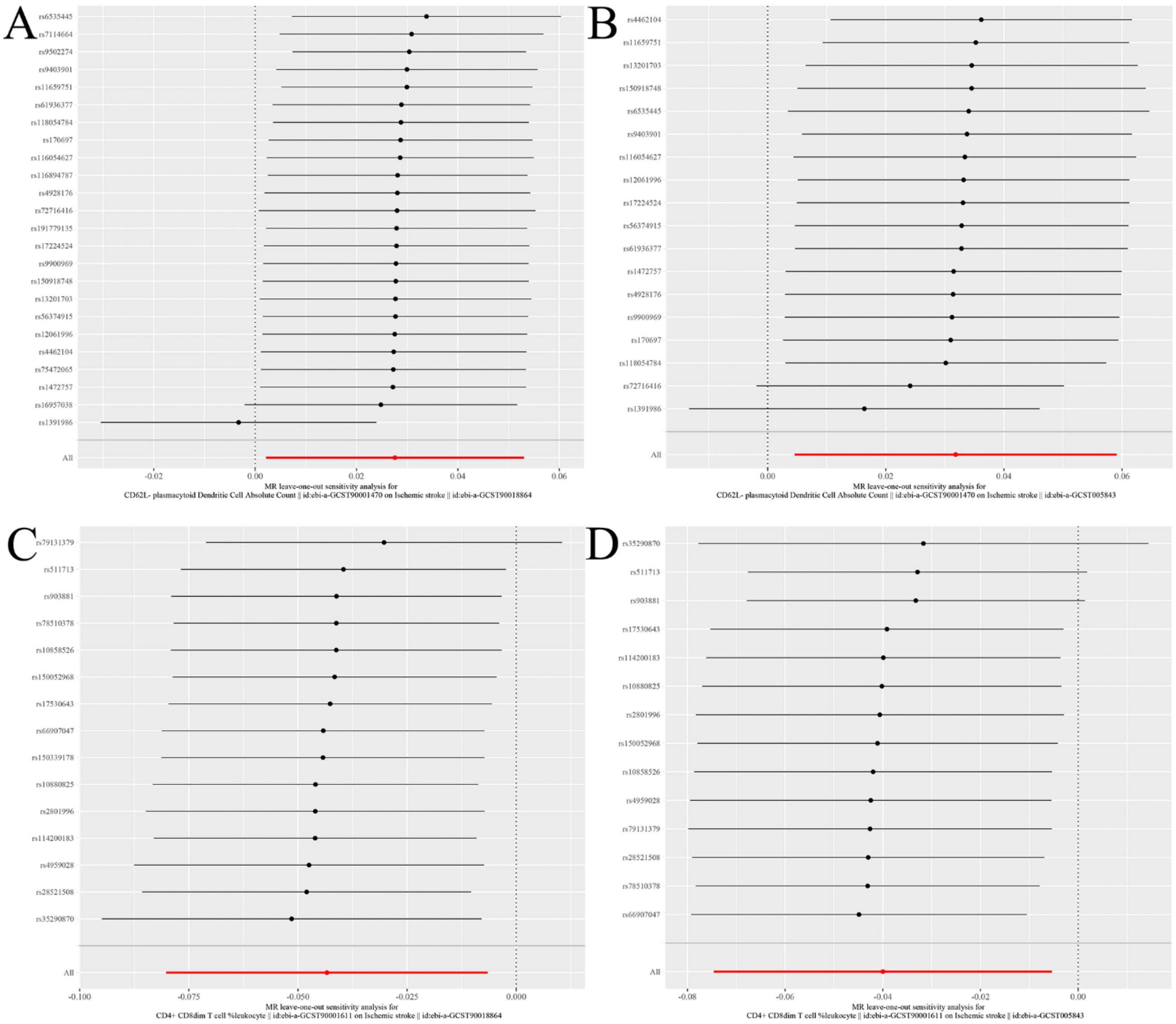

Supplementary Figure 3. Leave-one-out sensitivity analyses of the causal effects of immune cell traits on IS risk. **(A)** *CD62L- plasmacytoid Dendritic Cell Absolute Count* on IS risk based on discovery dataset. **(B)** *CD62L- plasmacytoid Dendritic Cell Absolute Count* on IS risk based on validation dataset. **(C)** *CD4+ CD8dim T cell %leukocyte* on IS risk based on discovery dataset. **(D)** *CD4+ CD8dim T cell %leukocyte* on IS risk based on validation dataset.

procedure code

```
if(T){
  rm(list=ls())
  options(scipen=200)
  path<-'E:\\'
  setwd(path)
  result.dir<-'immune cell traits-ischemic stroke'
  if(!file.exists(result.dir)){dir.create(result.dir)}
  setwd(result.dir)
  path<-getwd()

  info<-read.csv(file='20230921 TwoSampleMR.csv')
  exposure<-info[which(info[,'author']=='Orr<U+00F9> V'),'id']
  exposure.names<-info[which(info[,'author']=='Orr<U+00F9> V'),'trait']
  outcomes<-c('ebi-a-GCST005843','ebi-a-GCST90018864')
  outcomes.names<-outcomes

  library(TwoSampleMR)
  library(data.table)
  library(phenoscanner)
  library(dplyr)
  library(meta)
  library(ggsci)

  file_names=c("1-datasets",
```

```
            "2-OR",
            "3-heterogeneity",
            "4-pleiotropy",
            "5-leave-one-out",
            "6-funnel plots",
            "7-scatter plots",
            '8-forest plots',
            '9-meta-analyses',
            '10-SNP annotation',
            '11-confounders processing'
  )
  path_files=c()
  for (file_name in file_names){
    path_file=paste0(path,"\\",file_name,'\\')
    if(!file.exists(file_name)){dir.create(file_name)}
    path_files=cbind(path_files,path_file)
  }
#####1-datasets#####
    exp<-extract_instruments(outcomes=exposure,p1 =5e-8,clump=TRUE,access_token = NULL)
    exp$exposure<-paste0(exposure.names[match(exp$id.exposure,exposure)],exp$exposure)

    splitnum<-ceiling(nrow(exp)/100)
    index<-matrix(1:(splitnum*100),ncol=splitnum)
    PSdata<-{}
    for(i in 1:ncol(index)){
      snps<-na.omit(exp[index[,i],'SNP'])
```

```
PSdata<-rbind(PSdata,phenoscanner(snpquery=snps)$results)

}

write.csv(PSdata,file='PhenoScanner_GWAS_result.csv')



vte<-extract_outcome_data(snps = exp$SNP,outcomes =outcomes,proxies = FALSE,maf_threshold = 0.01,access_token =NULL)



keywords1<-list(a=c('Hypertension','Self-reported hypertension','Type 2 diabetes Hypertension Coronary artery disease combined case analysis','Vascular or heart problems diagnosed by doctor: high blood pressure'),

          b=c('Coronary artery calcified atherosclerotic plaque 90 or 130 HU threshold in type 2 diabetes',

              'Diabetes diagnosed by doctor','Diabetes mellitus type 1','Eye problems or disorders: diabetes related eye disease',

              'Illnesses of siblings: diabetes','Insulin-dependent diabetes mellitus',

              'Medication for cholesterol, blood pressure or diabetes: insulin','Self-reported diabetes','Self-reported type 1 diabetes',

              'Severe diabetic retinopathy in type 1 diabetes','Started insulin within one year diagnosis of diabetes','Type 1 diabetes',

              'Type 1 diabetes autoantibodies','Type 1 diabetes combined control dataset','Type 2 diabetes',

              'Type 2 diabetes Hypertension Coronary artery disease combined case analysis','Type 2 diabetes nephropathy','Type II diabetes',

              'Type II diabetes adjusted for BMI'),

          c=c('Blood pressure smoking interaction','Current tobacco smoking','Ever smoked','Former smoker',

              'Light smokers, at least 100 smokes in lifetime','Maternal smoking around birth','Nicotine initiation smoking',

              'Past tobacco smoking','Smoking status: current','Smoking status: previous','Tobacco smoking: ex-smoker'),
```

```
d=c('Alcohol consumption','Alcohol consumption in current drinkers','Alcohol dependence','Alcohol intake frequency',

  'Alcohol intake versus 10 years previously','Alcohol usually taken with meals','Alcoholism',

  'Reason for reducing amount of alcohol drunk: doctors advice'),

f=c('Body mass index','Body mass index adjsuted for physical activity in males','Body mass index adjusted for physical activity','Body mass index adjusted for smoking','Body mass index adjusted for smoking in females','Body mass index adjusted for smoking in males','Body mass index ajusted for physical activity in females','Body mass index females','Body mass index in female non-smokers','Body mass index in females','Body mass index in females greater than 50 years of age','Body mass index in females less than or equal to 50 years of age','Body mass index in male non-smokers','Body mass index in males','Body mass index in males greater than 50 years of age','Body mass index in males less than or equal to 50 years of age','Body mass index in non-smokers','Body mass index in physically active females','Body mass index in physically active indivdiuals','Body mass index males','Obesity body mass index'),

g=c('Age started oral contraceptive pill'),

h=c('Coronary heart disease'),

i=c('Cause of death: aortic valve disorder, unspecified','Cause of death: endocarditis, valve unspecified','Nonrheumatic aortic valve disorders'),

j=c('Self-reported transient ischaemic attack'),

k=c('Cause of death: stroke, not specified as haemorrhage or infarction','Self-reported stroke','Stroke, not specified as haemorrhage or infarction','Vascular or heart problems diagnosed by doctor: stroke')

)

names(keywords1)<-c('Hypertension','diabetes','smoking','Alcohol','BMI','oral contraceptive','Coronary heart disease','valve','transient ischaemic attack','stroke')

keywords1.snp<-unique(PSdata[which(PSdata[,'trait']%in%unlist(keywords1)),'snp'])

delete.snp<-vte[which(vte[,'SNP']%in%keywords1.snp),'SNP']

keywords1.snp.list<-matrix('',ncol=length(keywords1),nrow=length(delete.snp))

for(i in 1:length(keywords1)){

  inter.snp<-intersect(unique(PSdata[which(PSdata[,'trait']%in%keywords1[[i]]),'snp']),vte[,'SNP'])

  if(length(inter.snp)!=0){keywords1.snp.list[1:length(inter.snp),i]<-inter.snp}

}
```

```
colnames(keywords1.snp.list)<-names(keywords1)

write.csv(keywords1.snp.list,file='SNPs related to confounders.csv')

if(length(delete.snp)!=0){vte<-vte[-which(vte[,'SNP']%in%delete.snp),] ;exp<-exp[-which(exp[,'SNP']%in%delete.snp),]}

define.snp<-c('rs1391986')

if(length(which(vte[,'SNP']%in%define.snp))!=0){vte<-vte[-which(vte[,'SNP']%in%define.snp),] ;exp<-exp[-which(exp[,'SNP']%in%define.snp),]}

define.snp.PSdata<-PSdata[which(PSdata[,'snp']%in%define.snp),]

write.csv(define.snp.PSdata,file='define.snp.PSdata.csv')


output<-file(paste0(path_files[1],"snps removed.txt"), open = "wt")

sink(output, type = "message")

mydata <- unique(harmonise_data(exposure_dat=exp,outcome_dat=vte,action= 3))

sink(type = "message")

close(output)

remove.info<-as.matrix(read.table(paste0(path_files[1],"snps removed.txt"),sep='\t',quote=""))

remove.snps<-unlist(strsplit(grep('^rs',remove.info,value=T),', '))

if(length(remove.snps)>0){mydata<-mydata[-which(mydata[,'SNP']%in%remove.snps),]}

rownames(mydata)<-1:nrow(mydata)


res <- mr(mydata)

res<-res[which(res$method%in%c('MR Egger','Inverse variance weighted') & res$id.outcome%in%outcomes),]

f<-data.frame(mydata$beta.exposure^2/mydata$se.exposure^2)

colnames(f)<-""

write.csv(exp,file='exp.csv')

write.csv(f,file=paste0(path_files[1],'f.csv'))
```

```
  write.csv(mydata,file=paste0(path_files[1],'mydata.csv'))

#####2-OR#####
  OR<-generate_odds_ratios(res);length(which(OR[,'pval']<0.05))
  write.csv(OR,file=paste0(path_files[2],'OR.csv'))
  OR1<-OR[which(OR$pval<0.05 & OR$method=='Inverse variance weighted'),]
  inter.exposure<-OR1[duplicated(OR1$exposure),'exposure']

#####3-heterogeneity#####
  het <- mr_heterogeneity(mydata)
  het$I2<-round((het$Q-het$Q_df)/het$Q*100,0)
  het$I2[which(het$I2<0)]<-0
  write.csv(het,file=paste0(path_files[3],'het.csv'))

#####4-pleiotropy#####
  pleio <- mr_pleiotropy_test(mydata);length(which(pleio[,'pval']<0.05))
  write.csv(pleio,file=paste0(path_files[4],'pleio.csv'))

#####5-leave-one-out#####
  for(i in 1:length(exposure)){
    tab1<-mydata[which(mydata[,'id.exposure']==exposure[i]),]
    for(j in 1:length(outcomes)){
      tab<-tab1[which(tab1[,'id.outcome']==outcomes[j]),]
      if(nrow(tab)==0){next}
      single <- unique(mr_leaveoneout(tab))
      if(nrow(tab)>100){height=24}else{height=7}
```

```
      cairo_pdf(paste0(path_files[5],gsub('[\\]|[?]|[>]|[<]|[|]|[/]|[*]|[:]','-',exposure.names[i]),'-
',outcomes.names[j],'.pdf'), family="serif",width = 8, height = height)

      print(mr_leaveoneout_plot(single))

      graphics.off()

    }

  }


#####6-funnel plots#####

  res_single <- mr_singlesnp(mydata)

  for(i in 1:length(exposure)){

    tab1<-mydata[which(mydata[,'id.exposure']==exposure[i]),]

    for(j in 1:length(outcomes)){

      tab<-tab1[which(tab1[,'id.outcome']==outcomes[j]),]

      if(nrow(tab)==0){next}

      cairo_pdf(paste0(path_files[6],gsub('[\\]|[?]|[>]|[<]|[|]|[/]|[*]|[:]','-',exposure.names[i]),'-
',outcomes.names[j],'.pdf'), family="serif",width = 7, height = 7)

      print(mr_funnel_plot(mr_singlesnp(tab)))

      dev.off()

    }

  }


#####7-scatter plots#####

  for(i in 1:length(exposure)){

    tab1<-mydata[which(mydata[,'id.exposure']==exposure[i]),]

    for(j in 1:length(outcomes)){

      tab<-tab1[which(tab1[,'id.outcome']==outcomes[j]),]
```

```
      if(nrow(tab)==0){next}

      cairo_pdf(paste0(path_files[7],gsub('[\\]|[?]|[>]|[<]|[|]|[/]|[*]|[:]','-',exposure.names[i]),'-',outcomes.names[j],'.pdf'), family="serif",width = 7, height = 7)

      print(mr_scatter_plot(res,tab))

      dev.off()

    }

  }



##########8-forest plots #####

  for(i in 1:length(exposure)){

    tab1<-mydata[which(mydata[,'id.exposure']==exposure[i]),]

    for(j in 1:length(outcomes)){

      tab<-tab1[which(tab1[,'id.outcome']==outcomes[j]),]

      if(nrow(tab)==0){next}

      if(nrow(tab)>100){height=24}else{height=7}

      cairo_pdf(paste0(path_files[8],gsub('[\\]|[?]|[>]|[<]|[|]|[/]|[*]|[:]','-',exposure.names[i]),'-',outcomes.names[j],'.pdf'), family="serif",width = 7, height = height)

      print(mr_forest_plot(unique(mr_singlesnp(tab))))

      dev.off()

    }

  }



#####9-meta-analyses#####

  if(length(inter.exposure)!=0){

    pfs<-list()

    for(i in 1:length(inter.exposure)){

      AG <- OR[OR$exposure ==inter.exposure[i]  & OR$method=='Inverse variance weighted', ]
```

```
    lnor<- log(AG[,"or"])

    lnuci<- log(AG[,"or_uci95"])

    lnlci<<- log(AG[,"or_lci95"])

    selnor<-  (lnuci-lnlci)/(2*1.96)

    pfs[[i]]=metagen(lnor,selnor,sm="OR",data=AG,studlab=AG$id.outcome)

    cairo_pdf(paste0(path_files[9],gsub('[\\]|[?]|[>]|[<]|[|]|[/]|[*]|[:]','-',exposure.names[i]),'.pdf'),
family="serif",width = 10, height = 7)

    meta::forest(pfs[[i]],rightcols = c("effect", "ci",'pval'),leftcols =
c("studlab","TE","seTE"),leftlabs=c("Study","TE","seTE"),digits=3,digits.pval=3)

    dev.off()

    pfs[[i]]<-as.data.frame(pfs[[i]])

   }

  }

 }
```
